# Sensation

**Published:** 1890-01

**Authors:** George W. Whitefield

**Affiliations:** Chicago


### SENSATION.[1]

BY GEORGE W. WHITEFIELD, M.D., D.D.S., CHICAGO.

(Concluded from vol. x. p. 733.)

It is the law of nerve-action that a sensation once transmitted is more easily passed over the same nerve-track and more readily affects the nerve-centres ever afterwards; and in proportion as the same sensation is repeated over the same track, not only is the transmission more easily accomplished, but all the reflexes are similarly affected; hence the dexterity acquired in any art by practice,—dexterity consists in well-organized reflexes, produced by the same sensations being passed over the same nerve-track till you might say the track is worn smooth. In other words, the habit is formed, either physical or mental.

The following, from a newspaper clipping, illustrates one phase of the subject: "The regular tracks of thought sometimes betray the speaker. Thus, one of our ministers, speaking, from the pulpit, of the late Horace Cooke, called him Horace Greeley, there being a well-worn track that united Horace and Greeley.

"And another spoke of the sufferings of our Lord in the garden of Eden, the tracks between garden and Eden being better worn than between garden and Gethsemane. When a mind is in ruins it often runs in these well-beaten tracks. A very eloquent Presbyterian divine, happening to use the word Peter in one of his sermons, paused, and said, 'Peter, Peter, pumpkin-eater.' This was the last time he was permitted to occupy the pulpit."

Then, again, subjected to the influences that have gone before of the same kind, the nerves are modified by the equally important influence of sensations of a different kind.

Let me speak here of a theory of memory. Some physiologists teach that nerve-cells are not originally gray, but that the gray tint is imparted to the cells that have been called into use, and that these cells are capable of receiving or responding only to such sensations as have passed over them, and might be compared to sheets of paper that are written on and filed away for future reference; and a good memory means that the librarian has filed away

---

[1] Read before the Chicago Dental Club, September 23, 1889.

these cells in systematic order, so that they are easily found when needed.

For convenience, I have spoken of the sensations as if there were simple sensations, while, in fact, such a phenomenon never occurs. There are, however, paramount sensations,—that is, of all the sensations coming to one of you at a given instant of time, there is always a paramount sensation, and it is the one your attention is called to, as you at the same instant of time are receiving sensations by way of all the senses, but the sense that your attention is called to is the sense that receives a sensation, that becomes a cognition, but even that sense is incapable of receiving more than one sensation at the same time.

One would naturally suppose that the paramount impression on the simple nerve-centres or the sensorium would result from acts of paramount importance, which is not necessarily the case, as attention and habit cause sensations of minor importance to produce results out of all proportion to their real value.

Why do we not profit by this fact when operating on sensitive teeth?

Our appreciation of all sensations, no matter by which of the five senses they are received, is a mental act, and the meaning attached to that sensation is biased by the mental habits of the person receiving it. The effect of sensory impressions on the reflex centres, as well as on consciousness, depends on a great variety of interacting causes.

Foster says, "All the information which can be gained by the eye is limited to the field of vision, and, provided that the relative positions of the sensations in the field of vision remain the same, the actual position of external objects might, as far as vision is concerned, be changed without our being aware of it. As a matter of fact, the field of vision, in one important particular, does not correspond to the field of external objects. The image on the retina is inverted; the rays of light proceeding from the object which by touch we know to be on what we call our right hand, fall on the left-hand side of the retina. If, therefore, the field of vision corresponded to the retinal image, the object would be upside-down, and seen on the left side. We, however, see it on the right hand, because we invariably associate the right-handed tactile localization with left-handed visual localization. That is to say, our field of vision, when interpreted by touch, is a reinversion of the retinal image."

We never hear anything just as the sound is produced. Take a person who never heard a telephone, and let him apply the ear-piece to his ear; how natural for him to be deceived with regard to the evidence of his sense of hearing! In fact, without bringing other senses to assist, it is hard to localize the sound, and it is the same with regard to all our senses.

Our accumulated experience enables us to form a sense-judgment of what has taken place, yet how often are we deceived!

It takes a very appreciable length of time for a sensation to be conveyed from the periphery to the nerve-centres, where it takes another appreciable length of time for the pulse of excitement to take place in the nerve-centres, and another space of time for the motor impulse to be carried to its appropriate organ. But if an appreciable length of time is required for a sensory impulse to become a conscious sensation, a still longer period of time is required for that sensation to pass away, as is illustrated in the rapidly-revolving point of fire. It appears to be a circle: before one sensation has had time to fade, the point is back where it started.

Every one of you know how the sense of sight will play you false on a very clear or very dull day. Any object that you are not familiar with is liable to deceive you with regard to its size, unless there is something near of a known size to compare it with. Men standing near an object that is taller than we expect appear to be much shorter than they are.

Our love of art (the grand and beautiful) depends largely on the fact that many things may be suggested by the few. In this way the eyes appeal to our imagination. A picture is more or less perfect as the artist is capable of making it appear to contain more than is really on the canvas. A caricature depends entirely on what is suggested,—and does not music do the same?

Without the several senses the human mind would remain a blank forever, as all sensations are received by way of the five senses,—that is, so far as our present knowledge goes. I would like to dip into the probable but uncertain ground of psychology, but my paper will necessarily be too long if I give but a superficial glance at my subject,—sensation.

No one sensation could impart much to our consciousness if received only for the first time; but when different sensations are reproduced numbers of times, and the recorded impressions are compared with themselves and with each other, there is established the superstructure of memory, ideas, and perceptions, the arrangement, development, and particular use of which constitute what is termed culture.

Professor Alexander Bain, the noted Scotchman, says that "knowledge begins with differences. We do not know anything of itself, but the difference between it and something else." Consequently, the person is most cultivated in music who can perceive the most difference in time, pitch, etc.; so in a general sense those most cultivated perceive the most difference in all impressions received,—while he is wise who is capable of profiting by them, and formulating these impressions into ideas, and using them as occasion may require. These defined qualities we call mind.

I have been speaking of sensations received by the nerve-centres from without, as rays of light falling on the retina cause a consciousness of light. We become conscious of something touched,—vibrations in the air (unless we accept the substantialists' theory of the entity of sound) convey to our consciousness the sense of sound,—we hear.

Minute particles of matter floating in the air coming in contact with the lining membrane of the nose, we have the sensation of smelling; and other particles mingled with the oral secretions coming in contact with the gustatory or taste bulbs, there is passed by the nerves to the sensorium the sense of taste.

Under the influence of multiplied sensations the nerve-centres become modified in their action, so that after a while a slight impulse is sufficient to arouse as much action as a stronger one could do at first. You can each demonstrate for yourselves that by irritating the optic nerve by pressure on the eye, you will see light. If the irritation comes from a blow, you are liable to see stars.

Sensations become incorporated into the very substance of the nerve-structure, so that one sensation is capable of calling out indefinite numbers of other sensations or memories of other sensations, as the smell of fish cooking, coming from some kitchen, may arouse memories of some exploits with the rod and line. You perhaps recall some shady nook where you have angled for bass. You can even hear the water ripple as it eddies around a stump that juts out into the stream, and perhaps you recall the balmy fragrance from the wild apple-blossom overhead, hearing again the clear whistle of the bobolink as he perches on a reed across the stream.

All these sensations may be caused by the commonplace odor from a kitchen range. Nor is it at all necessary that there should be first a sensation received from the afferent nerves to start a-going all this train of sensations, as the mind is capable of initiating the first impulse and all succeeding impulses.

These centrally initiated impulses are what we, as dentists, need to know a great deal about to manage intelligently many of our patients, for there are many—women especially—who are not trained mentally, and who think that there is nothing less true than that one cannot believe the evidence of the senses.

Attention plays an important part in the phenomena of sensation and perception. A sensation that would pass unnoticed while the attention is engaged becomes apparent as soon as the diversion ceases. Attention renders one acutely sensible of what would pass unnoticed under ordinary circumstances.

You probably all know of good operators who have among the public the reputation of being harsh, solely because they fail to profit by this fact. The employment of mental anæsthetics is more than profitable,—it is humane. Our reputation is largely measured by the mental training we give our patients. For nothing is more true than that a painful operation can be rendered bearable (as we might say) by proper mental handling before and during the operation.

Do not accuse me of being a metaphysician, but think of the fact that we are incapable of receiving more than one sensation at one time, and that attention determines what that sensation shall be. A juggler engages your attention, and does his tricks before your very eyes without you seeing them.

An operator can, by controlling the attention of the patient, perform painful operations with but little inconvenience to the patient. This can be done without the unseating of the patient's reasoning power by hypnotism, provided the operator can inspire the patient with confidence, and to do that he must have confidence in himself, besides possessing an amount of sympathy, a strong will, and a thorough knowledge of his business. He brings into play his individuality, and accomplishes the task, as the hypnotist would say, by suggestion.

Attention determines the quality of the sensations we receive.

If you will carefully read the evidence offered in a court of law of some occurrence witnessed by a number of people, all of whom testify under oath, trying to be truthful in their statements, you will find a great diversity of statements, each one perhaps equally true, and stated as each one saw the occurrence, modified by the temperament, surroundings, and mental habits of the person. Each one would be impressed with what to that person was the paramount sensation.

Among your acquaintances you can recall persons who always catch those sensations which are ridiculous,—they see the funny side of life; while there is old Longface who taints the very air with melancholy, seeing yawning graves even in street excavations; he believes that all the world is in league against him. Then there is the man who sees suspicious actions in every one he meets. Compare this man's mental habits with the hearty, whole-souled individual who sees the silver lining in life's blackest storm-clouds. He rises above all ills, and says he is "glad to see you" in a manner that convinces you that he means just what he says.

How true this remark, which I heard Talmage make,—"The world is the color of the glasses we wear." The dyspeptic views the world through colored glasses. We expect to find him on the negative side of the question "Is life worth living?" while the solution of the conundrum depends upon the condition of his liver.

We are constructed much like a piano. Raise the cover of the instrument and halloo into it, and the sound sent back to you will be produced by the strings that are tuned to the same pitch as your voice, while all the rest will remain silent; so with our mental instrument: we tune it to catch the impressions we wish to receive. We may be unconscious of the act in this regard, as our inherited propensities, our surroundings, and our associations may have determined the class of impressions that we shall receive before we are capable of taking charge of our own education. But he is not worthy of the name of man who will make this excuse for continuing mental habits that are contrary to his judgment or wish. A man can make himself what he wishes to be if he will: apparent physical impossibilities will melt away to a large extent. We receive just what we have trained our minds to receive. On this principle a man is just what he will make himself, and he is not well balanced mentally who cannot make himself what he wishes to be.

This, I admit, is a broad statement, but science will bear me out in it.

There is a trite old adage, "Evil communications corrupt good manners." For, what we perceive is governed almost entirely by what we train our minds to receive, as the interpretation of all sensations is a mental act. This being true, as can be proven, what more simple or easy to comprehend than this,—we are all free moral agents? A man is what he makes himself.

Excuse this diversion. I am drifting from my subject.

I was saying that habit and attention largely determine what we see and hear, and that what we see and hear is only imperfectly received, as only one of our senses can receive a sensation that shall become a conscious sensation at the same instant of time, so that all other sensations are lost or so imperfectly received that we are conscious of a vague something that we strive to give a meaning to, and, if we have a similarly recorded sensation to compare it with, we may, by using our reasoning powers, give the sensation its appropriate meaning.

I stated that a single sensation is capable of calling up other sensations or memories of other sensations, and that the sensation that shall start a-going this wonderful power of memory may be centrally initiated; that the mind is capable of initiating the first and all succeeding sensations, and that they are just as real as if they were received by way of the afferent nerves from the external world. You will all admit, as they are so tangible, that they often deceive even the most careful of us, and how much more are they liable to deceive one whose mental balance is imperfect!

What I wish to speak of now are these centrally initiated sensations, and the danger of mistaking them, as so many do, for sensations of an external origin, for these pulses of nervous energy of cerebral origin are accepted as evidence of bodily conditions by a large portion of the community. People of the emotional temperaments have a pulse of action rising higher than occasion requires: an explosion of energy takes place when a mere pulse would be the expression in an ordinary person. These people allow the evidence of their senses to deceive them,—that is, they allow sensations of a cerebral origin to impress them just as they would those reaching the sensorium by way of the afferent nerves.

To illustrate: A lady sitting in my office, while I was using my electric motor, complained of a headache; as soon as I commenced to use my instrument she said the headache had ceased, as electricity always cured her headache. Now, the electric current did not pass near her, and it was impossible for her to receive any benefit from it: it was a freak of the imagination. This person interpreted the assumed evidence of her feeling by another set of emotional evolutions. This person lacked what many others do whose ideas of their bodily conditions are the result of these double pulses of centrally-excited nerve-action. Some of these creatures, where mental discipline is at a big discount, are apparently made up of highly-explosive material; they go into ecstasies of delight or are plunged into the depths of melancholy by occurrences that would go unnoticed by a person possessed of a well-balanced temperament. Such a person is always having queer or horrible sensations in that portion of the body to which his or her attention is directed from any cause,—most often in the back and head. And why should there not be strange sensations in these nerve-centres, where such prodigal waste is constantly going on, where violent explosions take the place of throbs of nervous force? These people give to their sensations names which usually correspond to nothing that they have any previous knowledge of.

Emotional people are apt to be "marked" with certain antipathies; certain things which they eat will have peculiar action on them. I do not think that this can be easily combated; it is incorporated into the very structure of the individual. I know a cultured young lady of rare courage and splendid mind, a leader and queen among women, who will almost lose her self-control if a cat approaches her, while she will not hesitate to mount and ride the most fractious horse with absolute confidence. In nothing else does she know what fear means.

My argument is not against emotional people; it is only that they shall know how to distinguish between emotions centrally initiated and those from the periphery. For, what a dull, cold world this would be should all emotional people or all emotions be banished from it! Emotions are the bonds that unite the home; they are the main-spring of the genus. What would inspire us to prolonged exertion save our emotions? Was it not the enthusiasm (emotions) aroused in loyal breasts by our patriotic and soul-inspiring war-songs that won us our men and victories in the late national unpleasantness? "We are coming, Father Abraham, ten hundred thousand strong." Did not these words carry truth with them? How they thrilled even the dried bones of apathy! The emotions nerved the delicate man to endure hardships where the less emotional man with stronger physique succumbed,—on the march, in the field, and (where the emotions manifest their greatest power for physical weal or woe) in the hospital: where hope was gone men died from trivial injuries, while the enthusiasm inspired by success or the hope of seeing loved ones would restore men who were literally shot to pieces.

Persons possessed of good education, "smart" people, people whom we naturally look to, expecting from them great things, those whose mental poise we would not think to question, are often guilty of accepting memories of past sensations as evidence of present conditions; for this is what these centrally initiated sensations are,—they are distinct memories of sensations that have been passed over the nerve-track and have worn the track smooth. As I stated before, in speaking of habit, by irritating the optic nerve by electricity or a blow we perceive light, irritating the auditory nerve produces sound, and interfering with our pet, the fifth pair, after it has been for some time conveying painful sensations, will often cause it to transmit those sensations again, even when the irritation is not of a painful character.

You probably all will have patients tell you, as I have had them tell me, that they always have a tooth filled twice,—once in the dental chair, and then afterwards their mind calls up to consciousness memories of the operation, and they live the time over again, going through the whole operation, experiencing all the pleasant (?) sensations.

These centrally initiated sensations may be distinct memories, as I stated before, or vague shadowy memories of sensations partly forgotten, or but partly or imperfectly received, or they may be a combination of many sensations that have passed over the nerve-track, not being exponents of any sensation.

As an illustration of the first class, I will give the experience of a young lady of undoubtedly good education, but of slightly æsthetic tendencies. At the institution where she was a student she with a number of others formed a circle for testing their ability to endure shocks from an induction coil. As the result of the shock she received, she had a violent headache, and each day after that, when they performed experiments in the class, she would have a recurrence of the headache, although nothing would induce her to take hold of the handles again. Now, while it may be that the first headache was caused by the shock (which I doubt), each succeeding attack must have been caused by these centrally excited sensations, as the electric current would follow the conductors as accurately as the water will flow through the water-pipes.

With the teeth, it is not always that an operation is painful, but attention renders one hypersensitive.

Here it is again. Attention determines what we shall become conscious of. The degree of consciousness of a sensation depends on the degree of attention paid to it. John may be sick while contemplating the unfinished task, while base-ball means changed conditions and occupied attention. The more our attention is called to bodily ills the more we exaggerate them, while if something drives self out of our minds we begin to improve. Does the metaphysician tell the dyspeptic to eat this and not to eat that? No: it would defeat the object. He tells the patient to go about his ordinary employment and let the stomach alone. The stomach when left alone will usually take care of itself. Poverty would cure many of the incurable invalids that can receive no good from their physicians.

It is a well-known fact that many people are capable of self-hypnotization,—that is, they surrender their entity to an idea, just as to the hypnotized patient a suggestion of an idea makes that idea real. Bennett mentions the case of a butcher who wished to place a heavy piece of meat on a hook above his head; he slipped, the hook caught him by the arm, and he remained suspended. He was taken down half-dead, his sleeve was cut open, and, although he complained of great suffering, as soon as the arm was exposed it was found to be absolutely intact: the hook had only penetrated the coat-sleeve.

This novel case has a parallel in many bed-ridden invalids who have been ill for years. Yes, I mean they are sick. These patients received the class of sensations that they train their nerves to receive, which in turn so modifies their structure that organic structural change takes place. Such patients are more to be pitied than those who are ill from structural change without the modified nerve-action, as in one case when the cause is removed the nerves resume their normal action, while in the other the pathological condition is due to perverted nerve-action, produced in turn by the will of the patient governed by attention,—victims of ideation due to suggestion.

I know that I am laying myself open to harsh attacks by those who are really sick, but such sickness is due to the same causes that produced burns, etc., in the following subjects of hypnotic experiments, except that these patients are their own hypnotists.

Charcot, at the Salpêtrière has produced the effect of burns on the skin by means of suggestion.

During a *séance* Bourron and Burt, professors of the Rochefort School, have produced blood-sweat by suggestion. On one occasion, after one of these experimenters hypnotized the subject, he traced his name on the subject's arm with the blunt end of a probe, and told the patient that at four o'clock he would go to sleep, and blood would issue from the lines on his arm. The subject went to sleep at the time, and the letters appeared in bright red lines with an occasional minute drop of blood. *This* subject in a spontaneous attack of hysteria commanded his arm to bleed, and soon after the cutaneous hemorrhage just described was displayed.

It is also well authenticated that any part of the body of a hypnotized patient may change in volume, simply owing to the fact that the patient's attention is fixed on that part. This influence may be exerted in hyperexcitable subjects by the simple phenomenon of ideation on the vaso-motor centres.

Disease in emotional people is not as often imaginary as due to the imagination. It is the physiological excitement of peripheral sensations till habit is formed and disease results, and it can be combated by the nerves that caused it.

Expectation—faith—will cause that change to take place. It may be faith in the physician, faith in prayer, change of climate or surroundings: the patient's attention must be drawn from his trouble.

It is not the object of this paper to attack any beliefs,—it is simply to call attention to laws of nerve-action.

I will state, in closing this long paper, that persons of highly sensitive nervous temperament do not possess that quality of level-headedness requisite to put their sensations into the mental balance, testing them to know for a certainty whether they are initiated at the cerebral end of the nervous system or are received by way of the senses, doubting even the evidence of their senses until their reason confirms the sensations. In fact, unless such persons are possessed of mental attributes that even a person of a wide mental range is often deficient in,—viz., the ability to weigh thoroughly all sensations received, giving just value to all by the aid of the reason, rejecting the spurious or imaginary sensations,—in fact, subjecting all sensations to the crucial test of mental analysis,—if, I say, such persons do not possess those mental abilities and use them, they will constantly be liable to deception.

